# Retraction: Long non-coding RNA PSMA3-AS1 enhances cell proliferation, migration and invasion by regulating miR-302a-3p/RAB22A in glioma

**DOI:** 10.1042/BSR-2019-1571_RET

**Published:** 2023-03-01

**Keywords:** cell proliferation, glioma, miR-302a-3p, PSMA-AS1, RAB22A

This article is being retracted from *Bioscience Reports* at the request of the authors following receipt of a notification from a reader, alerting the Editorial Office to similarities between images found in this paper and those in papers by unrelated authors. The Figure 6E RAB22A and B-actin blots appear to be the same as the Figure 5b RAB14 and GAPDH bands from Xu et al. 2020 (https://doi.org/10.1186/s13046-020-01556-4), and the Figure 3C U251 and LN229 blots also appear to be the same (after decompression) to the Figure 3A HS68 and U87 blots from Feng et al. 2019 (https://doi.org/10.1186/s13046-019-1070-x). The authors are unable to correct the article and wish to retract the article. The Editor-in-Chief and Editorial Board agree with the retraction.

